# Recent Advances and Future Challenges in Pancreatic Cancer Care: Early Detection, Liquid Biopsies, Precision Medicine and Artificial Intelligence

**DOI:** 10.3390/jcm12237485

**Published:** 2023-12-04

**Authors:** Lois A. Daamen, I. Quintus Molenaar, Vincent P. Groot

**Affiliations:** 1 Department of Surgery, Regional Academic Cancer Center Utrecht, UMC Utrecht Cancer Center and St. Antonius Hospital Nieuwegein, 3584 CS Utrecht, The Netherlands; l.a.daamen-3@umcutrecht.nl (L.A.D.);; 2 Division of Imaging and Oncology, University Medical Center Utrecht Cancer Center, Utrecht University, 3584 CS Utrecht, The Netherlands

## Abstract

The incidence of pancreatic ductal adenocarcinoma (PDAC) is rising. While surgical techniques and peri-operative care have improved, the overall survival for PDAC remains poor. Thus, novel and bold research initiatives are needed along the spectrum of clinical care, a few of which will be discussed in this article. Early detection is crucial, with specific high-risk groups possibly benefiting from targeted screening programs. Liquid biopsies (such as circulating exosomes, tumor DNA, or tumor cells) offer promise as multifunctional biomarkers for early detection, treatment guidance, and recurrence monitoring. Precision medicine is being explored via targeted therapies for actionable mutations, such as PARP inhibitors for BRCA mutations, and immunotherapy strategies. Artificial intelligence (AI) is emerging as a powerful tool in medical imaging, biomarker discovery, genetics research, and treatment planning, and it can aid in diagnosis, treatment selection, and patient monitoring. However, its associated challenges include ethics, data security, algorithm reliability, and validation. Collaborative efforts between medical professionals, researchers, and AI experts are vital for unlocking AI’s potential to enhance pancreatic cancer care. In conclusion, despite the challenges, advancements in liquid biopsies, precision medicine, and AI offer hope for enhancing the diagnosis, treatment, and management of pancreatic cancer.

## 1. Introduction

The incidence of pancreatic ductal adenocarcinoma (PDAC) is continuing to rise, and it is projected to become the second leading cause of cancer-related death by 2030 [1]. In recent decades, marked improvements in surgical technique and peri-operative care have reduced the rates of severe morbidity and mortality after pancreatic resections [2,3]. Moreover, muti-drug regimens such as FOLFIRINOX (fluorouracil, leucovorin, irinotecan, and oxaliplatin) or gemcitabine combined with nab-paclitaxel have augmented the available treatment options and strategies [4,5]. However, despite all these encouraging developments, the overall survival remains poor across all stages of PDAC, especially when compared to the significant progress made in other solid malignancies. To further the cause of patients with PDAC, novel and bold research initiatives are needed along the spectrum of clinical care, a few of which will be discussed in this Editorial.

## 2. Early Detection and Screening

Pancreatic cancer is typically diagnosed at a late stage, with symptoms often indicating advanced disease [6]. As such, only 10–20% of newly diagnosed PDAC patients are eligible for a potentially upfront curative resection. Early detection is regarded as the holy grail of pancreatic cancer research by many researchers, in that it would meaningfully impact survival outcomes.

### 2.1. Screening

Pancreatic cancer is currently not suitable for population-based screening due to its low incidence and the lack of accurate, affordable, and non-invasive screening tests. However, the screening of specific groups within the population who are at a higher risk of developing pancreatic cancer has the potential to be both cost-effective and improve survival. As such, there is a need to identify high-risk patient groups in whom surveillance could lead to early detection and intervention via preventive treatment or surgery in the pre-symptomatic stages of the disease.

Currently, patients with hereditary cancer predisposition syndromes and familial pancreatic cancer represent high-risk groups who should be considered for screening surveillance [7]. These include Peutz–Jeghers syndrome, BRCA gene mutations, hereditary pancreatitis, familial atypical multiple mole melanoma syndrome (FAMMM), Lynch syndrome, familial adenomatous polyposis (FAP), and several other less well-known syndromes [8,9]. The other significant group eligible for screening surveillance are patients diagnosed with either intraductal papillary mucinous neoplasms (IPMNs) or mucinous cystic neoplasms (MCNs) of the pancreas [10]. These potentially pre-malignant cystic neoplasms can progress to lesions with high-grade dysplasia, or even to invasive pancreatic cancer. The difficulty of surveilling and managing these patients stems from recognizing distinctive features that indicate high-grade dysplasia. The detection of these “worrisome” features should lead to timely resection, while only observing lesions with low-grade dysplasia should prevent unnecessary pancreatic resection [11].

### 2.2. Novel Tools for Early Detection

An ideal tool for early detection would consist of a non-invasive, repeatable and low-cost test, with high specificity and high sensitivity for the detection of early-stage resectable PDAC or even pre-malignant high-grade dysplasia [6]. Early-stage pancreatic cancer is notoriously difficult to detect via conventional imaging, eliminating the concept of widespread CT surveillance. However, recent advances in combining the fields of radiomics (mining specific data from imaging scans) and machine learning have demonstrated promising results [12]. At Johns Hopkins, for instance, the FELIX project aims to train artificial intelligence (AI) using deep neural networks to train computers in order to help radiologists detect tumors in CT scans at an early stage with promising results [13].

For the surveillance of pancreatic cysts, a recent study showed that serum carbohydrate (CA) 19-9 sampling was not predictive of high-grade dysplasia or pancreatic cancer [14]. Moreover, CA 19-9 monitoring in patients with pancreatic cysts caused harm by shortening surveillance intervals and increasing the likelihood of unnecessary surgery. As an alternative for CA 19-9, the surveillance of pancreatic juice for biomarkers and/or gene mutations is being researched extensively. Due to the advances in next-generation sequencing, the mutational analysis of cystic fluid is gaining in clinical relevance. For instance, the detection of *KRAS* in cyst fluid has shown >80% sensitivity and specificity for diagnosing IPMNs and MCNs [15]. However, for the aspiration of cyst fluid, invasive procedures such as endoscopic ultrasound with fine-needle aspiration (EUS-FNA) and/or endoscopic retrograde cholangiopancreatography (ERCP) are needed. Unfortunately, these procedures are often technically demanding and not without complications.

## 3. Liquid Biopsies

Apart from early detection, novel biomarkers are required to guide treatment selection, determine the treatment response and predict recurrence after surgery. CA 19-9 is currently the most widely employed blood-based protein marker for patients with PDAC. However, it can be also elevated in patients with extra-pancreatic malignancies and benign conditions. Furthermore, approximately 6% of the Caucasian population and 22% of the African American population in the USA are Lewis antigen-negative, meaning that they do not produce CA 19-9 [16].

Liquid biopsies have exhibited promise as a novel and multifunctional biomarker in multiple malignancies [17,18]. The term ‘liquid biopsy’ is used to describe several emerging technologies focused on extracting biomarkers from bodily fluids such as saliva, urine and pancreatic fluid, but mainly blood [19]. The most studied genetic and biological materials include circulating tumor cells (CTCs), extracellular vesicles (e.g., exosomes), circulating tumor DNA (ctDNA), microRNA (miRNA), and cell-free RNA. Liquid biopsies possess distinctive advantages in contrast to conventional tissue biopsies. These benefits encompass their minimal risk, convenient sample collection, their more accurate depiction of tumor heterogeneity, and their ability to perform serial samplings. The latter facilitates real-time, dynamic, and longitudinal analyses, thereby providing comprehensive and prolonged surveillance information. Due to previously mentioned advances in next-generation sequencing and DNA/RNA amplification, enthusiasm regarding the potential application of liquid biopsy tests is rapidly growing.

### 3.1. Liquid Biopsies as Screening Tool

Multiple studies have evaluated the promising utilization of liquid biopsies as a screening tool for detecting early-stage PDAC. However, the sensitivity of ctDNA for detecting early-stage resectable pancreatic cancer varies substantially, from 30 to 60% [20]. Sensitivity for later-stage locally advanced pancreatic cancer (LAPC) and metastatic disease is much higher at 70–95% [21]. These discrepancies indicate that, with the current techniques, detecting ctDNA in the blood during the early stage of cancer remains challenging. Although less extensively studied, exosomes might prove more suitable as a screening tool; this is because (1) pancreatic cancer cells are often exocrine and exosomes are continuously released in the bloodstream, and (2) exosomes have a longer half-life than ctDNA. Research on CTCs as a screening tool in PDAC is limited. While the detection rates of CTCs in late-stage PDAC are substantial, the yield of CTCs in early-stage PDAC is limited. It is possible that smaller tumors do not yet shed whole tumor cells, or that the liver filters most CTCs at the early onset of the disease.

### 3.2. Liquid Biopsies to Guide Treatment and Monitor Recurrence

Multiple studies have revealed that the presence of both ctDNA and CTCs in the immediate pre- and post-operative period is a strong predictor for worse progression-free and overall survival [22,23]. In this way, liquid biopsies could help select patients and guide both neoadjuvant and adjuvant systemic therapy. For example, one study demonstrated that CTCs with mesenchymal-like features predict poor survival only in patients who either experience a delay in initiation or are not in receipt of adjuvant therapy [24]. This suggests that a delay in adjuvant therapy could potentially provide the residual systemic disease with a window of opportunity to recover from the surgical insult. In addition, the post-operative monitoring of ctDNA and CTCs can predict the recurrence of disease up to 3–6 months prior to detection via conventional imaging surveillance, generating the potential to treat recurrence at an early stage with a lower tumor burden [22,23,25]. Although the body of evidence on exosomes is smaller, a high-quality study showed both the strength of exosome DNA as a peri-operative predictor for survival and a surveillance predictor of recurrence [26].

The clinical use of the analysis of somatic and germline mutations of pancreatic cancer is steadily increasing. For this purpose, ctDNA could be used to identify potentially actionable mutations such as DNA damage response mutations, which predict benefits to poly(ADP-ribose) polymerase (PARP) inhibitors or platinum chemotherapy [27]. Furthermore, CTCs could provide insights into drug resistance mechanisms and elucidate the underlying mechanisms of the therapeutic response by examining samples from patients undergoing diverse treatments. Furthermore, these CTCs can serve as a basis for the generation of organoids and patient-derived xenograft (PDX) models.

Despite the soaring optimism regarding the use of liquid biopsies in pancreatic cancer care, several critical limitations currently hamper its whole-scale integration into clinical practice [17]. Importantly, patients with PDAC often have very low levels of circulating genetic material (often in the range of 0.1% mutated DNA relative to wild-type DNA), necessitating expensive ultrasensitive and reproducible approaches for eventual clinical application [15]. As such, the rate of false negative results remains high, especially in patients with localized disease. Moreover, despite all the promising preliminary results described above, there are no clinical trials yet demonstrating that liquid biopsy-based treatment actually improves outcomes in pancreatic cancer patients. However, encouragingly, recent trials in colon and lung cancer have proven that liquid biopsies possess the potential to meaningfully impact patient survival [28,29]. In the near future, currently ongoing prospective and interventional clinical trials will determine whether treatment decisions based on liquid biopsies can enhance care for patients with PDAC.

## 4. Precision Medicine

In recent decades, our understanding of the mutational and immune landscape of PDAC has greatly improved. However, compared to several other tumor types, the immunotherapy and/or treatment of actionable mutations have yet to meaningfully improve outcomes for pancreatic cancer patients [30,31]. Current efforts aiming to further unravel the biology and genetics of PDAC will hopefully lead to the development of an effective precision medicine approach, including targeted and immune-based therapies [32].

### 4.1. Targeted Therapy

Due to large-scale sequencing programs in patients with PDAC, several actionable mutations have been discovered; these are defined as a genetic aberration for which a specific targeted therapy exists [33]. Patients with a pancreatic tumor with or without an actionable mutation have comparable survival. However, when a matched, precision-based therapy is administered to the matched actionable mutation, survival can nearly double [33]. For PDAC, the most well-known examples of targeted therapies are *BRCA* mutations receiving a PARP inhibitor or patients with microsatellite instability and high status receiving an immune checkpoint inhibitor [27]. Although effective, the number of PDAC patients that harbor these mutations is relatively low.

The oncogenic *KRAS* mutation is prevalent in >90% of PDAC patients, making it the most obvious target for precision-based treatments. However, the development of *KRAS*-specific inhibitors has proven challenging due to a lack of molecular binding sites for potential drugs [34]. However, the field of drug development has witnessed significant advances, leading to the introduction of pharmacological tools specifically designed to target mutations in the *KRAS* gene. In patients with wild-type *KRAS* tumors (around 10%), the most common potential actionable mutations include *BRAF* and *NTRK*. However, the research of treatment for these mutations in PDAC is currently mostly limited to pre-clinical studies, with limited data on humans. Another way to classify PDAC tumors is based on gene expression-based subtyping. Multiple studies have revealed that specific molecular subtypes might predict worse or improved survival outcomes [35]. A subsequent trial in metastatic pancreatic cancer showed that the radiographic response to modified FOLFIRINOX differed significantly based on the gene expression-based subtype [36]. The clinical implications are currently being investigated in a phase II trial randomly assigning patients to either a modified FOLFIRINOX and gemcitabine/nab-paclitaxel group (NCT04469556—PASS-01 trial). In both groups, integrated molecular profiling, patient derived organoid establishment, drug testing sensitivity and biomarker discovery is performed.

### 4.2. Immunotherapy

To date, the effect of immunotherapy in PDAC has largely been disappointing, except for the 1–2% of patients with microsatellite instability and high tumors who can be treated with PD-1/PD-L1 inhibitors (immune checkpoint blockade) [37]. This is possibly explained by the micro-tumor environment of PDAC, with cancer cells closely surrounded by macrophages and fibroblasts. Novel strategies include enhancing T-cell activity with new vaccines, adoptive cell therapies, and novel checkpoint blockade targets [32]. Additional approaches currently being explored involve exploiting dendritic cells to enhance the tumor-specific T-cell response and to target the micro-tumor environment network of protective fibroblast and macrophage stroma. As with targeted therapy, an enhanced translational understanding of PDAC will hopefully lead to an increase in precision-based and immunotherapy-based therapies able to treat this systemic and refractory disease.

## 5. Artificial Intelligence

Recent developments in data science, particularly the emergence of AI, hold promise with regard to addressing the ongoing challenges present in pancreatic cancer care. These innovative technologies aim to (1) improve diagnostic accuracy and efficiency, (2) aid biomarker development, (3) unravel genetic complexities, (4) support the development of data-driven personalized treatment strategies, and (5) enable streamlined healthcare processes through the automation of routine tasks in the foreseeable future. AI offers a unique opportunity to integrate multi-omic data into one comprehensive test, potentially revealing novel patterns for prognostication and response assessment. While these advancements have substantial promise, they also come with certain challenges that warrant thoughtful consideration, including ethical aspects, legal frameworks, data security, integration into clinical practice, and quality assurance [38].

The application of AI is particularly notable in medical imaging, encompassing both radiology and pathology. AI algorithms have demonstrated their capacity to assist healthcare practitioners in the analysis of medical images. The accurate detection of anomalies in pathology slides and radiological imaging can contribute to the detection and monitoring of pancreatic cancer [39,40]. This is particularly helpful given the high workload of clinicians. In addition, the disease’s complexity often requires extensive expertise, which is not always available. Furthermore, AI has the potential to mitigate interobserver variability through automation and algorithmic standardization. Nonetheless, ensuring the reliability and consistency of algorithms across diverse patient groups, imaging modalities and software platforms remains a challenge that mandates thorough validation and refinement.

Tailoring treatment plans to individual patients is a cornerstone of modern oncology. AI’s capacity to analyze diverse datasets, encompassing molecular profiles, treatment responses, and patient outcomes, has the potential to empower oncologists to explore personalized therapeutic strategies [41]. AI tools also exhibit promise in analyzing complex biological datasets, expediting biomarker and genetic factor discovery. This facilitates the efficient analysis of large-scale genomic data, assisting researchers in uncovering relevant genetic variations and interactions. Nevertheless, interpreting the complex genetic landscape of pancreatic cancer demands collaboration between AI specialists and geneticists, to ensure accurate insights that lead to actionable outcomes. Through predictive modelling and real-time data-analysis, AI is anticipated to aid healthcare teams in data-driven decision-making regarding treatment plans, patient monitoring, and the adaptation of interventions as needed [42]. By predicting how individual patients are likely to respond to various treatments, AI is expected to optimize treatment regimens, ultimately reduce unnecessary adverse effects and augment the likelihood of successful outcomes. This is particularly relevant for pancreatic cancer care, given the heterogeneous nature of the disease and its propensity for resistance to conventional anti-cancer therapies.

As AI continues to evolve within the healthcare sector, several challenges need to be addressed. The ethical considerations of integrating AI within healthcare, concerning topics such as patient privacy and transparency in decision-making processes, alongside algorithm trustworthiness and post-implementation quality assurance, require thoughtful considerations. Ensuring the reliability, interpretability, and ethical application of AI-driven solutions is paramount. Additionally, the validation of AI models via diverse and representative datasets is essential to avoid bias and ensure beneficial outcomes for all patients [43]. The scarcity of the large, well-curated datasets required for the development of sophisticated AI techniques, such as deep learning, constitutes a substantial challenge that mandates comprehensive collaboration among research groups on a global scale [44]. Furthermore, weighing the benefits of automation with the necessity for personalized (human) patient care and clinical expertise poses a challenge that demands careful consideration. For this purpose, a balance between innovation, patient well-being and data security needs to be achieved. Collaborative efforts between medical professionals, researchers, and AI experts will be essential in overcoming these challenges and unlocking the full potential of AI in enhancing medicine, including pancreatic cancer care.

In conclusion, recent advances in AI have introduced vast potential regarding the augmentation of pancreatic cancer care across various domains. From refining medical imaging to aiding in biomarker discovery and genetics research, AI offers valuable insights and efficiencies. However, the journey ahead involves addressing challenges related to the validation, ethics, legal frameworks, and the clinical implementation of explainable and trustworthy algorithms. To ensure that AI continues to contribute safely and effectively to improving pancreatic cancer care over time, quality assurance processes should be framed. Nevertheless, a well-established synergy between AI and medical expertise can optimize patient care, paving the way for the more efficient and effective management of pancreatic cancer.

## 6. Conclusions

In conclusion, pancreatic cancer remains a lethal disease with the poorest survival rate of any solid malignancy. Alarmingly, the incidence and mortality rates for pancreatic cancer have continued to rise in the past two decades, in stark contrast to other common cancer types. However, remarkable advances have been made in the molecular understanding of pancreatic cancer; clinical trials employing novel multi-drug chemotherapies have exhibited promising survival outcomes, and pancreatic surgery is saver than ever before. Due to these advances in multi-modality care, treatment indications and options have significantly expanded, even for patients with locally advanced, oligometastatic or recurrent disease. On the other hand, these improvements add to the complexity of providing multi-disciplinary treatment for pancreatic cancer patients.

This multi-modality care opens the door to new strategies and innovations related to, for instance, AI, neoadjuvant and adjuvant therapies, robotic surgery, the application of liquid biopsies, and immune and precision therapy. To achieve truly personalized care for pancreatic cancer, we require improved tools for patient and treatment selection, superior biomarkers and radiomics to measure treatment response, and novel therapies that target tumor-specific genetic vulnerabilities. This Special Issue hopes to address these current advances and future challenges in the field of pancreatic cancer research in order to achieve profoundly improved outcomes for patients.

As such, we cordially invite you to submit your original or review articles reflecting clinical or translational research to this Special Issue of *JCM* entitled “Pancreatic Cancer: Recent Advances and Future Challenges”. Beforehand, we would like to thank all reviewers for their insightful comments and help in further improving the manuscripts included in this Special Issue, and the *JCM* team for their support. Additionally, we wholeheartedly thank the authors for their valuable and high-quality contributions, which will shape this Special Issue and will help further the cause for patients with pancreatic cancer.
